# Purple Tea (Camellia sinensis var. assamica) Leaves and Obesity Management: A Review of 1,2-Di-Galloyl-4,6-Hexahydroxydiphenoyl-β-D-Glucose’s (GHG) Potential Health Benefits, and Future Prospects

**DOI:** 10.7759/cureus.75055

**Published:** 2024-12-03

**Authors:** Arun Kumar Balasubramaniam, Ashmitha Elangovan, Maheen Abdul Rahman, Subhendu Nayak, Aleksander Richards, Durga Swain

**Affiliations:** 1 Department of Pharmaceutical Sciences, College of Pharmacy and Health Sciences, Texas Southern University, Houston, USA; 2 Department of Zoology, PSGR Krishnammal College for Women, Coimbatore, IND; 3 Department of Pharmacy, PA College of Pharmacy, Mangalore, IND; 4 Probiotics and Supplements, Vidya Herbs USA, Bunnell, USA; 5 Research and Development, Vidya Herbs USA, Bunnell, USA; 6 Pharmaceutical Biotechnology and Microbiology, Vidya Herbs USA, Bunnell, USA

**Keywords:** anti-obesity, extracts, natural products, purple tea, weight loss

## Abstract

Purple tea (*Camellia sinensis* var. *assamica*) is a distinct variety of *Camellia sinensis* known for its bioactive compounds, including caffeine, catechins, and a unique compound called 1,2-di-Galloyl-4,6-Hexahydroxydiphenoyl-β-D-Glucose, (GHG) found predominantly in purple tea leaves, which shows potential in obesity management. Studies have indicated that these bioactive compounds play a significant role in reducing BMI and body weight among obese patients. This review focuses on how GHG impacts body weight and BMI in obese patients. A comprehensive literature review was conducted using Science Direct, Semantic Scholar, Wiley, PubMed, and Google Scholar databases up to 2024. The search employed both single keywords (e.g., 'purple tea', 'GHG', 'obesity') and multiple keyword combinations (e.g., 'purple tea and obesity', 'GHG and weight loss') related to purple tea, GHG, obesity, BMI, and clinical studies. The database search yielded 246 articles, with 173 articles retained after removing duplicates and studies published before 1999. This systematic approach aimed to gather comprehensive data on the phytochemistry, pharmacology, and potential therapeutic applications of purple tea. The investigation revealed that GHG operates through multiple mechanisms, such as inhibiting pancreatic lipase to reduce fat absorption, suppressing adipogenesis and lipogenesis, and preventing fatty tissue formation. Clinical investigations demonstrated significant reductions in BMI, waist circumference, and body weight among individuals consuming purple tea extracts with high GHG levels. Additional metabolic benefits include increased energy expenditure, improved insulin sensitivity, and enhanced glucose metabolism regulation. While more comprehensive research is needed to fully elucidate the optimal dosage and long-term effects, current evidence suggests that GHG from purple tea could be a valuable natural intervention in the multifaceted approach to obesity management.

## Introduction and background

Obesity has emerged as a critical global health challenge, characterized by its complex interplay with numerous comorbidities such as type 2 diabetes, cardiovascular diseases, and certain malignancies. While traditional weight management strategies encompass lifestyle modifications, dietary interventions, and pharmacological treatments, there is growing interest in exploring natural, plant-based alternatives that offer potentially safer and more holistic approaches to weight control [1,2]. Traditional herbal medicine is applied as a nutritional supplement, and traditional medicine is used globally. Historically, plants were used as fragrances, cosmetics, and in cooking. They still serve as the main source of traditional medicine for curing various diseases [3]. This has bridged modern drug discovery development with ancient traditional knowledge through the identification of several compounds with known or novel mechanisms from high-throughput screening and combinatorial chemistry followed by analytical application of these hits that either mimic or enhance the observed clinical activity [4].

The leaves of *Camellia sinensis* (tea plant) have been utilized over countless years as a popular beverage with potential health benefits. Purple tea (*Camellia sinensis var. assamica*) distinguishes itself from other herbal remedies through its unique phytochemical profile, particularly its rich anthocyanin content and the novel polyphenolic compound 1,2-di-Galloyl-4,6-Hexahydroxydiphenoyl-β-D-Glucose (GHG) [5,6]. Unlike conventional anti-obesity medications that often carry high risks of side effects and potential abuse, purple tea presents a promising natural intervention with multifaceted mechanisms of action. The bioactive compounds in purple tea, especially GHG, demonstrate remarkable potential in weight management. Preliminary studies reveal that GHG can effectively modulate key metabolic pathways by inhibiting pancreatic lipase activity and influencing adipogenesis at the molecular level. This molecular mechanism suggests a targeted approach to weight reduction that goes beyond simple caloric restriction [6]. Furthermore, the synergistic effects of anthocyanins, catechins, and other polyphenols amplify the tea's metabolic benefits, with research indicating improvements in insulin sensitivity and reduction of inflammatory markers associated with obesity [7-9]. Probiotic yogurt fortified with purple tea containing prebiotic polyphenols enhanced the number of good gut-modifying bacteria while decreasing infections. As a result, the study shows that probiotic microbes and purple tea bioactive substances in the unique symbiotic yogurt work well together to improve the health of gut commensal bacteria [10].

**Figure 1:**
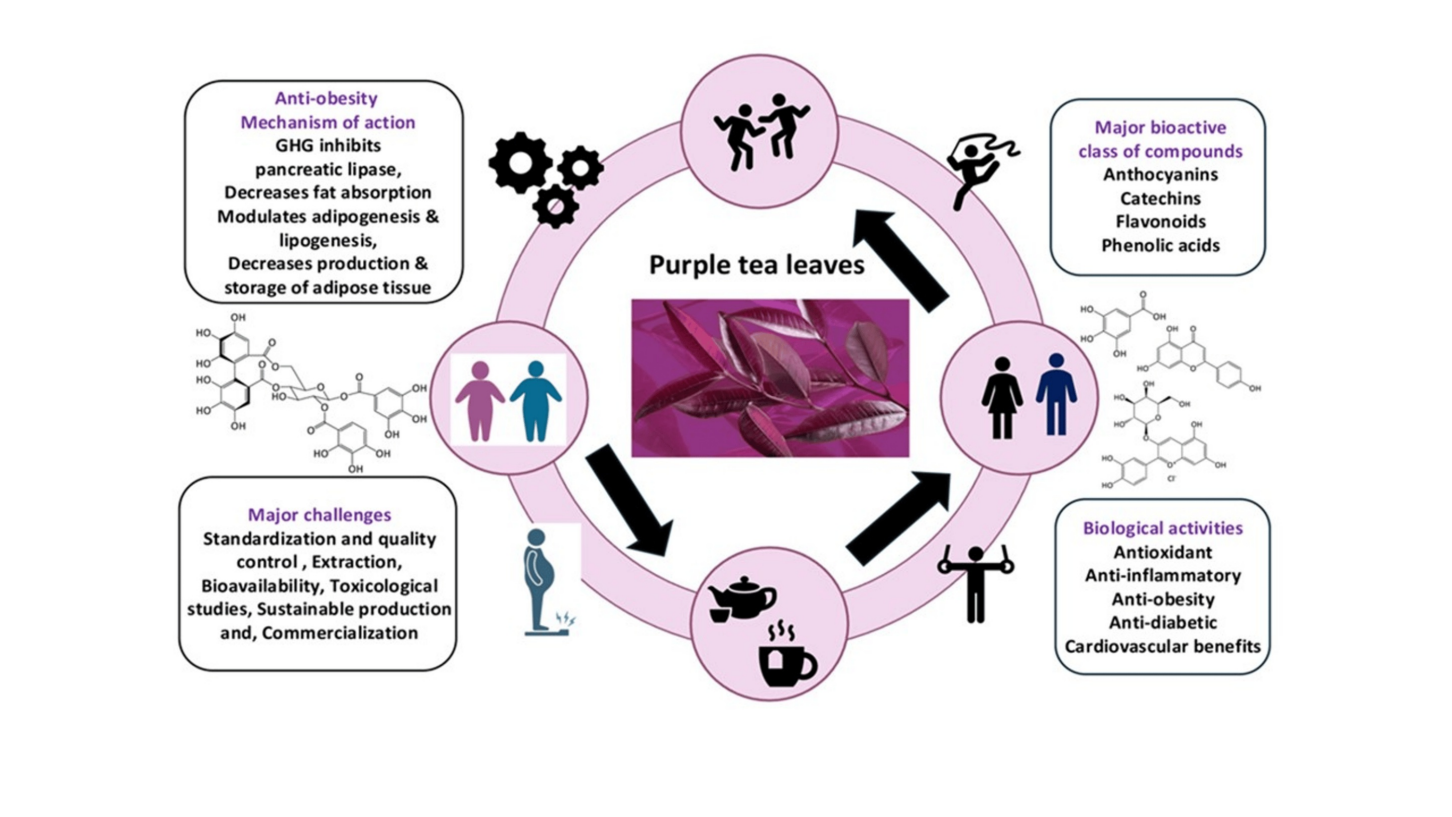
Purple tea of its potential in weight management, biological activities, and prospects. The image is illustrated by the author Arun Kumar Balasubramaniam.

Clinical investigations have provided compelling evidence of purple tea's efficacy. Subjects consuming GHG-rich purple tea extracts demonstrated significant reductions in body mass index (BMI), waist circumference, and overall body weight [5,11]. This empirical support positions purple tea as a potentially transformative natural intervention in obesity management. This review aims to comprehensively examine the scientific evidence surrounding purple tea's role in weight reduction, with a particular focus on the unique properties of GHG and its complementary bioactive compounds. By exploring its botanical characteristics, chemical composition, traditional uses, and potential therapeutic applications, this paper seeks to illuminate the promising landscape of purple tea as a functional food with significant health-promoting potential (Figure 1).

## Review

Materials and methods

**Figure 2:**
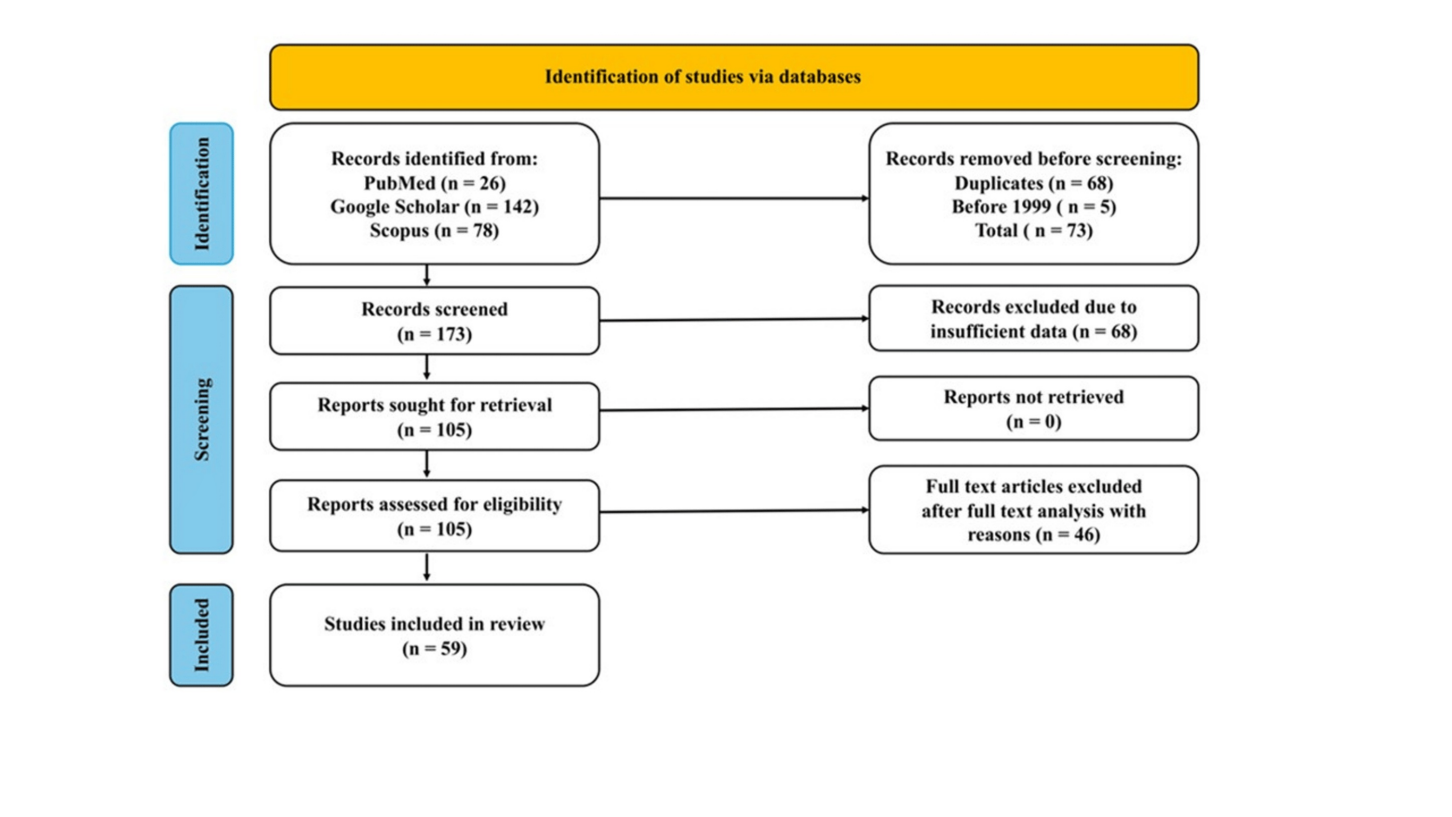
PRISMA flowchart for study selection.

A literature review was conducted using the Preferred Reporting Items for Systematic Reviews and Meta-Analyses (PRISMA) reporting guidelines (Figure 2). These three databases provided 246 articles (26 from PubMed, 142 from Google Scholar, and 78 from Scopus). In total, 73 records were removed before screening (68 duplicates and 5 from before 1999), leaving 173 records for screening. The analysis then excluded 68 articles due to insufficient data. Of the remaining 105 reports assessed for eligibility, 46 were excluded based on the context of full-text articles. Finally, 59 studies were included in the systematic review. Until 2024 PubMed, Google Scholar, and Scopus databases were searched systematically using specific keywords like "Purple tea," " Camellia sinensis var. assamica," "Polyphenols," "Anthocyanins," "GHG," "Phytochemistry," "Species," "Varieties," "Biological activities," "Obesity," "Antioxidants," "Diabetics," "Traditional uses," "Cardiovascular," "BMI," "Anti-inflammatory," "Side effects," "Synergistic effects," "Exercise," "Interactions," "Clinical studies," "Comparison," "Therapeutic potential," "Combinations," "Commercial products," "Toxicity," "Safety," "Challenges," "Limitations," "Formulations," "Gut microbiota," and "Gut health.

Overview of botanical characteristics of purple tea

Camellia is a genus of over 325 species, primarily from the highlands of southeast India. The tea, which is the most important taxonomically and commercially of all Camellia species, differs in this ability to make it brew. Some other Camellia species are used as ornamentals [12]. Purple tea is a distinct type of *Camellia sinensis var. assamica* that has leaves with purple coloration. Its young shoots can range in color from deep purple to reddish-purple, depending on the cultivar and growth conditions. These colors are due to high levels of anthocyanins. Immature shoots, older leaves, and stems may have purple coloring. Purple tea plants grow as shrubs or small trees like any other kind of tea. They can reach heights of between 1-2 meters when cultivated but can be much taller if left to grow wild. This plant has numerous branches on its bushy appearance [13]. Leaves have an elliptical-lanceolate form with serrated margins while having a glossy upper surface and, at the bottom, could be lighter green or light purple [14]. The flowers grown by purple tea are white with yellow stamens (2-4 cm across). They show up either alone or three together on leaf axils. Like other teas, purple tea relies on a deep taproot system with numerous lateral roots for water absorption from deeper layers of soil where nutrients are also found. It takes longer for a purple tea to mature than it does for a green tea. In comparison to other green tea varieties, they exhibit more robustness in their adaptability, especially during times when there is drought and cold temperatures, such as those experienced in China, where it thrives naturally better than elsewhere [15]. Visually, the purple color of these special plants is not just delightful but because of a gene mutation that controls anthocyanin production. To be exact, activating flavonoid 3',5'-hydroxylase (F3'5'H) gene expression elevates delphinidin-based anthocyanins synthesis. Therefore, alongside their adaptability to diverse weather situations, this particular trait makes purple tea plants magnificent. They flourish in highlands (between 1500 and 2500 meters above sea level) and can survive extreme temperatures than most varieties of green tea plants. Their ability to grow slower than the green tea varieties and still be more resistant to environmental challenges such as drought and low temperatures is an attribute of their unique nature [13,16,17]. Despite this, purple tea leaves do perform photosynthesis even though they are dark in color. The function of anthocyanins is to act as a sunscreen by protecting the photosynthetic system against excessive light damage, particularly under high altitude conditions [18]. Certain cultivars of purple tea plants have shown better resistance against pests and diseases that normally afflict tea crops. This is partly due to the presence of high levels of flavonoids and other secondary metabolites [15]. Purple tea’s botanical attributes make it an exclusive addition to the world of teas, with aesthetic appeal and potential agronomic benefits.

Phytochemistry

**Figure 3:**
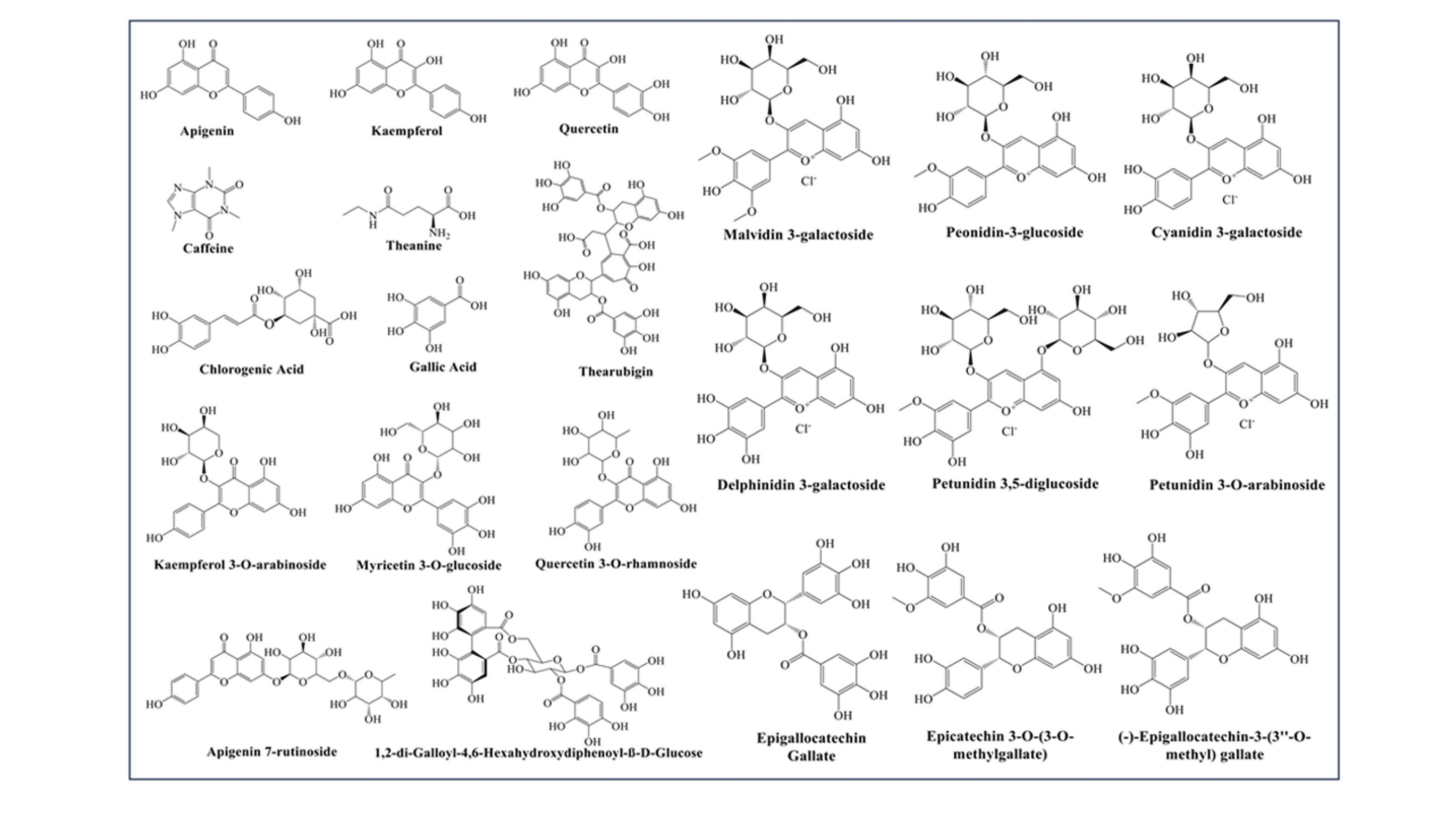
Structures of the major phytoconstituents identified in purple tea. The image is illustrated by the author Arun Kumar Balasubramaniam.

The phytochemistry of purple tea is complex, with various types of bioactive compounds such as anthocyanins, catechins, flavonoids, and phenolic acids being present. The typical purple coloration comes mainly from anthocyanin, which belongs to a group known as flavonoids. Prominent anthocyanins in purple tea include delphinidin-3-O-galactoside and cyanidin-3-O-galactoside (Figure 3). Other anthocyanins present included pelargonidin-3,5-diglucoside cyanidin-3-O-glucoside delphinidin, cyanidin, pelargonidin, peonidin, and malvidin, among others. These confer antioxidant properties on the tea and determine its color [19, 20]. Purple tea contains high levels of catechins, especially epigallocatechin gallate (EGCG), epicatechin gallate (ECG), and epigallocatechin (EGC). These compounds are known for their antioxidant and potential anti-cancer properties [16]. Purple tea contains quercetin, kaempferol, and myricetin glycosides. These glycoside compounds contribute to the tea's antioxidant capacity and potential health benefits [21]. It contains significant amounts of phenolic acids such as gallic, chlorogenic, and p-coumaric acid. Many health benefits are associated with these compounds. Tea plants contain a unique amino acid called theanine. It is believed to have cognitive-enhanced and stress-reducing potentials. Even though purple tea has lower caffeine content compared to black tea, it still has some caffeine like other teas. The bioactive compound GHG is unique to purple tea and has been found to have potent α-amylase inhibitory activity, suggesting potential anti-diabetic effects [21]. The phytochemical profile of purple tea varies according to growing conditions, processing methods, and tea leaf age. Purple tea is known for having higher total polyphenol content and antioxidant activity than green or black tea. These compounds give rise to possible health benefits in the form of antioxidants, anti-inflammatories, antidiabetic agents, and weight loss agents observed in purple tea. However, more research is needed to fully understand the bioavailability and efficacy of these substances in human health.

Comparison with other tea varieties

Purple tea is usually prepared in a similar way to green tea, with minimal oxidation performed to protect the anthocyanins, but sometimes it may be partially oxidized. Like its green counterpart, purple tea is also rich in catechins, mainly EGCG. Nevertheless, it has exceptionally high amounts of anthocyanins, especially delphinidin and cyanidin glycosides. It generally has less caffeine than other types of tea [19]. Purple tea showed higher total antioxidant activity than green and black teas because of the presence of both flavonoids and anthocyanins. For example, weight management potential, diabetes prevention, and cardiovascular health [16, 19]. Purple tea typically has a milder, smoother flavor than green tea, with delicate sweet notes. It contains a unique compound, GHG, with possible anti-diabetic characteristics not seen in other kinds of teas [21]. Green tea, on the other hand, undergoes minimal oxidation through boiling briefly to prevent enzyme action. Traditional teas are characterized by high levels of catechins, which give them elevated antioxidant power, and among them, green tea has the highest content. Also, purple tea has less caffeine than other teas, such as green tea. The research on it has been extensive regarding its potential benefits for cancer prevention, weight reduction, and cognitive function [22]. It is characterized by a grassy, vegetal taste and can be a little astringent [23]. On the other hand, green tea lacks anthocyanins and GHG, which are found in purple tea. The black teas are fully oxidized and, hence, have a dark color and robust flavor. It contains fewer catechins because of oxidation but more theaflavins and thearubigins than black tea. Purple tea sometimes contains lesser amounts of caffeine than black tea. Although it lacks the antioxidant activity of green tea, it does contain significant amounts of theaflavins and thearubigins [24]. Black teas may offer protection from heart disease and reduce some cancers’ incidence rates. It has strong flavors that are capable of being acrid taste-wise. Just like green tea, black tea lacks both high concentrations of anthocyanin and unique GHG, which exist in purple tea. Oolong, which falls between green and black teas, undergoes partial oxidation during its processing stage concerning its chemical composition levels; hence, this beverage features intermediate levels of catechins, theaflavins, and thearubigins. Compared to purple varieties, there is often higher caffeine content in oolong teas. Its antioxidant activity is generally comparable to that of green or black tea [25]. Such studies suggest possible applications against overweight-related disorders including diabetes mellitus. Oolong contains diverse flavors ranging from light flowery notes to deep roasted profiles depending on how much they are allowed to ferment, unlike green or black varieties. Like green and black teas, oolong tea does not have significant anthocyanin content.

Traditional uses

Purple tea, a cultivar developed in Kenya during the 1980s, is used in various traditional ways around its regions of origin. Purple tea (*Camellia sinensis var. assamica*) leaves can be prepared using several methods, with the most common being the standard hot brewing technique using water at 85-90°C for 2-3 minutes with 2-3g leaves per 240ml water. Alternative methods include Japanese-style brewing at lower temperatures (70-75°C) for shorter durations (1-1.5 minutes), cold brewing (6-8 hours at 4°C), and the traditional Kenyan method using boiling water with extended steeping (5-7 minutes) often accompanied by milk and sweeteners [5,19]. The brewing parameters can influence the extraction of bioactive compounds, including the characteristic GHG content [16]. Traditional applications include diabetes management, blood glucose regulation, and improved cardiovascular health, while scientific studies have pointed out potential anti-diabetic, anti-obesity, and cholesterol-lowering aspects. People use purple tea as a drink and flavoring agent in local dishes [26, 27]. Purple tea’s strong antioxidant content has become a frequently used component in traditional skin and hair care remedies [16]. In many parts of Africa, where it grows naturally, such products are commonly used for medicinal purposes or to maintain good skin. Purple tea was also used for ceremonial rituals by some communities, such as greeting ceremonies and celebrations [28]. Tea-growing countries have employed purple tea as a valuable cash crop, and extensive expertise has been utilized to develop value-added products. For food and non-food applications alike, purple tea flakes are rich in protein and minerals [29]. However, it is worth noting that even though purple tea is relatively new, many of its ‘traditional’ uses are still emerging and being integrated into local cultures with continuous scientific study supporting them. These unique qualities of purple tea, including its high levels of anthocyanins as well as its unusual taste, have contributed to its popularity both within traditional settings as well as among contemporary lifestyle consumers.

Biological activities

Antioxidant Activity

Purple tea can scavenge free radicals due to the abundant presence of polyphenols such as catechins and anthocyanins, which have significant antioxidant capabilities. For instance, research established that purple tea extracts had higher amounts of antioxidants compared to those from black and green teas. Tea’s many possible health benefits can be attributed to its antioxidant activity, which fights oxidative stress and damage from free radicals. In particular, the phenolic compounds in purple tea, like anthocyanins, have excellent properties for scavenging free radicals. The investigation showed that purple tea extract could effectively quench several ROS species, including superoxide anions, hydroxyl radicals, and hydrogen peroxides [16]. This makes it highly effective in fighting diseases caused by oxidative stress. That’s because purple tea has antioxidant compounds, particularly catechins, which have metal-chelating effects. This means that it binds to ions like iron and copper, thereby stopping them from being involved in free radical-generating processes. In this regard, Kerio et al. revealed high metal chelation activity in their purple tea extracts as part of their overall antioxidant profiles [19]. The lipid oxidation connected to many ailments has been decreased by the purple tea extracts. Several studies showed that purple tea polyphenols had significantly reduced malondialdehyde levels, a marker for lipid peroxidation in experimental models [21]. In another study, phenolic-rich extract from purple tea inhibited lipid peroxidation induced in egg yolk homogenate with an IC_50_=455 mg/L). It is essential to note that catechins and anthocyanins present in purple tea act synergistically as antioxidants. This implies that the antioxidant functionality of purple teas is known to exceed those of its parts, indicating complex interplay among its bioactive constituents [16]. The DPPH (2,2-diphenyl-1-picrylhydrazyl) and ABTS (2,2′-azinobis-3-ethylbenzothiazonline-6-sulfonic acid) IC_50_ value ranges were 25.27-166.47 μg/mL and 10.71-144.21 μg/mL respectively for the in vitro antioxidant activity of purple tea extract and isolated anthocyanins [30]. According to de Moura et al., at pH 4.5, anthocyanin-rich purple tea extracts exhibited about 73% inhibition against DPPH radicals, while at pH 10, they showed only about 39% [31].

Antidiabetic Activity

Several studies have shown that purple tea leaves have potential anti-diabetic properties. Its wide range of phytochemical profiles is promising for diabetes management and associated metabolic disorders. The research study reported GHG, a unique compound in purple tea that exhibited strong α-amylase inhibitory activity. This can help control rises in blood sugar levels after eating, which are caused by the slowing down of digestion of carbohydrates [21]. As an anti-diabetic agent, purple tea possesses tremendous antioxidant and anti-inflammatory potential. It is well-established that chronic inflammation and oxidative stress contribute much to the onset and progression of diabetes. Lai et al. reported that purple tea extract could mitigate oxidative stress related to diabetes by reducing inflammatory markers as well as enhancing antioxidant defenses [16]. The way glucose is metabolized has a significant influence on the composition of gut flora, which can be affected by the consumption of purple tea. As Tang et al. suggest, one such polyphenol from purple tea may have prebiotic benefits that improve metabolic health [32]. Purple teas significantly lowered α-glucosidase activities. In the study done by these researchers, it was observed that α-glucosidase had been inhibited by anthocyanin-rich fractions from purple tea, thus delaying carbohydrate absorption within the small intestine and thereby improving glycemic control. On top of this, da Silva et al. observed that other than polysaccharides, amylase was also inhibited by myricetin derivatives and kaempferol [33]. Similarly, cyanidins were found to be major players in this event, particularly regarding starch digestibility, therefore making purple tea preferable over other known teas for post-prandial hypo-glycemic effect. Tolmie et al., however, proposed that commercial purple teas with ellagitannins were more potent inhibitors against α-glucosidases than green teas and acarbose [34].

Antiobesity Activity

**Figure 4:**
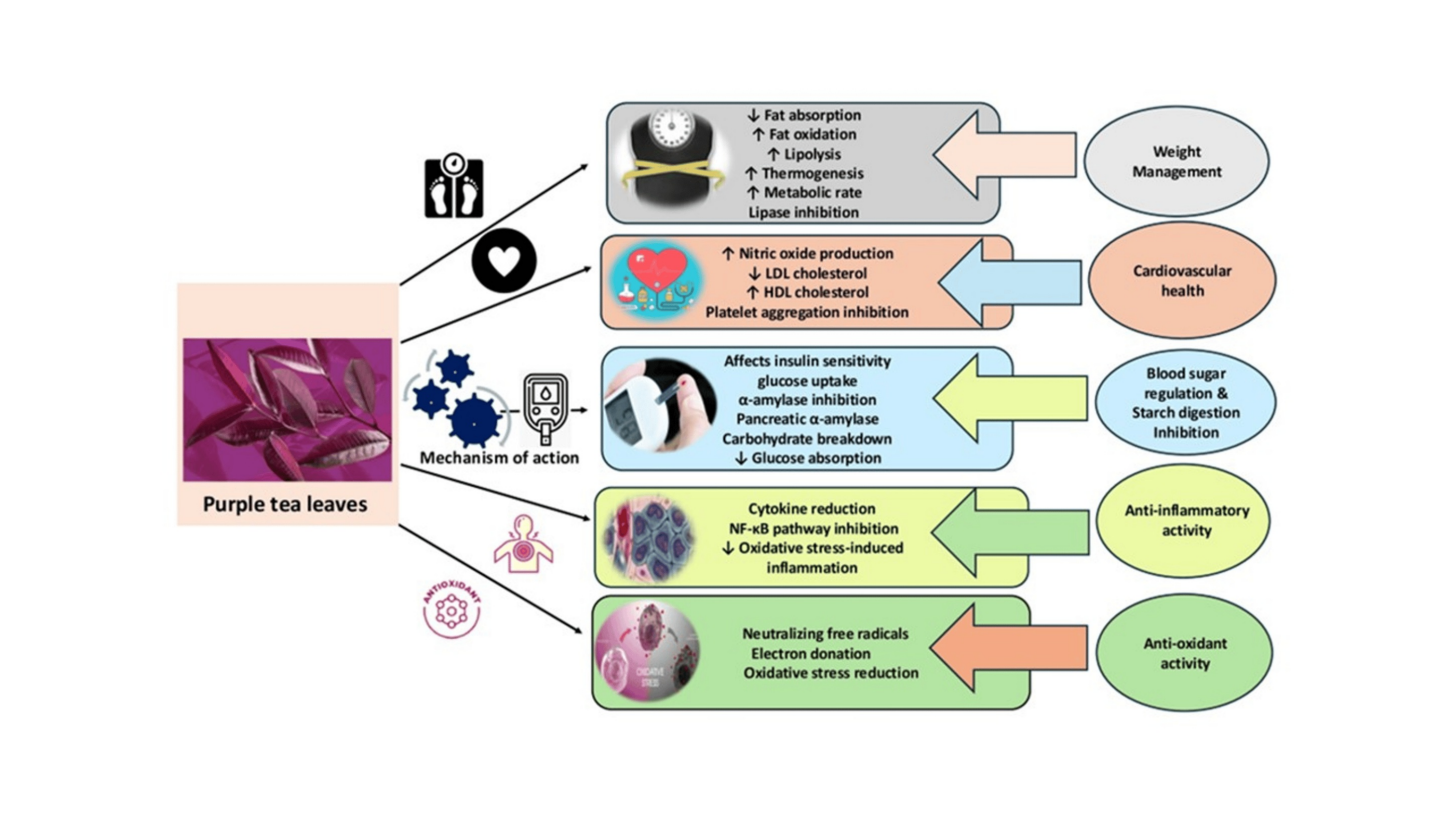
Mechanism of action of purple tea. The image was illustrated by the author Ashmitha Elangovan.

Purple tea is known for its potential to prevent obesity, specifically by checking on the influence of GHG. This hormone has potent inhibitory effects on pancreatic lipase, which is vital in fat digestion and absorption (Figure 4). Purple tea GHG was found to be a better inhibitor of lipase than EGCG, which is a famous green tea catechin, as shown by this research. For example, it can reduce fat absorption in the intestines, leading to weight loss [35]. Other researchers found out that adipogenesis prevented by purple tea’s GHG refers to the production of new fat cells. Its’ effect indicates how PPARγ and C/EBPα could be downregulated for GHG to inhibit preadipocyte maturation into mature adipocytes, hence controlling excessive body fat production [36]. GHG is known to increase lipolysis, which refers to the breaking down of stored fats. Also, they revealed that purple tea rich in GHG enhanced the level of hormone-sensitive lipase (HSL) production in adipose tissues, enhancing triglyceride breakdown and resulting in fatty acids that are used as energy [5]. GHG in purple tea has been shown to affect its metabolism. An experiment demonstrated by Chang et al. [37] indicated that GHG elevated uncoupling protein 1 expression in brown adipose tissue, possibly increasing thermogenesis and thus energy expenditure. Some studies suggest that the presence of GHGs in purple tea may have an impact on appetite control. There has been little scientific study into this aspect, but lower food intake was observed among animals nourished with green tea extract containing high levels of GHG. This result suggests a possible role for satiety signals [37]. Based on new findings, it can be concluded that GHG contributes to obesity by affecting gut microbiota composition. In addition, the studies established that the gut microbiota can be shaped by some classes of compounds, such as tea polyphenols, including GHG, allowing for weight reduction purposes. However, more research about purple tea and GHG is required, considering the existing information gap identified therein. Obesity is very often accompanied by low-level chronic inflammation. High contents of powerful anti-inflammatory agents, such as those found in purple tea extract rich in both GHG and other polyphenols, were claimed by researchers as being useful for alleviating inflammation linked to obesity itself. Nonetheless, it should be noted that actions against obesity are specifically attributed not only to GHGs themselves but also synergistic influences from several chemical compounds at once while playing an important role in this process. It was found that the bioactivity of purple tea can be improved by combining GHG with other polyphenols. Lai et al. [16] revealed that adding GHG to other polyphenols enhanced their overall bioactivity and hence health benefits.

GHG has been shown to affect glucose and lipid metabolism, which are usually deranged in obesity. Another notable finding of the study was that GHG present in purple tea suppressed α-amylase and α-glucosidase enzymes for better glycemic control. Additionally, patients who took purple tea extract had lower serum triglyceride levels than those who consumed other beverages [5, 21]. A trial on humans investigated the long-term effects of taking a drink containing an extract from purple tea leaves. Hence, compared to the placebo group, significant decreases were registered. They include body weight, BMI, and body fat percentage, among others [38]. Purple-leaf anthocyanin-rich tea has been shown to combat obesity as well as metabolic disorders. Nevertheless, it is still unclear whether consumption might protect against obesity and diet-induced metabolic disorders by altering gut flora [39]. The consumption of purple tea is associated with reduced weight gain and lowered body mass index [40]. A recent study also revealed that consuming four weeks’ worth of a beverage made from purple tea resulted in a decrease in key obesity indicators such as body weight, mass index (BMI), and fat mass when compared to baseline data only [9]. However, it should be noted here that not all studies assessing the potential anti-obesity benefits have included human clinical trials or experiments done on animals. Therefore, more clinical research is required to determine the full potential of purple tea and GHG as an anti-obesity drug and recommend suitable dosages.

Antiinflammatory Activity

According to the research study, Kenyan purple tea anthocyanins or coenzyme-Q10 can decrease CNS inflammation and thus reduce clinical manifestations associated with *Trypanosoma brucei rhodesiense* [41]. Inflammatory cytokine production was also reduced by extracts of purple tea. High anthocyanin content purple tea extracts significantly reduced TNF-α and IL-6 amounts in lipopolysaccharide-stimulated macrophages. These cytokines are crucial in starting and sustaining inflammation responses. The anti-inflammatory effect of purple tea is likely due to many compounds working together. This is because, according to the researchers, purple tea possesses many polyphenols that might contribute to its general anti-inflammatory function more than a single compound would [16]. The lyophilized extract of purple tea leaves taken in this study contained such compounds as flavan-3-ols and anthocyanins, which are capable of removal of free radicals like nitric oxide, peroxynitrite, and other reactive nitrogen and oxygen species [42, 43]. In addition to inhibiting pro-inflammatory transcription factors’ activation and reducing the levels of pro-inflammatory cytokines [44]. A recent study shows that most extracts from teas increase IL-6 expression in LPS-stimulated macrophages. However, all types of teas suppress the downstream translation of chemoattractant MCP-1 for immune cell trafficking. Inhibiting IL-6 expressions and TNF-α by all the teas were found to be dose-dependent in the prophylactic model, which suggests that they can serve as functional beverages for immune modulation (Table 1)[45].

**Table 1:** Biological activities of purple tea.

Author, Year	Mechanism of action	Biological activities
Kerio et al., 2012 [14]	Chelation of metal ions that catalyze oxidation	Antioxidant activity
Karori et al., 2007 [28]	Upregulation of antioxidant enzymes	
Joshi et al., 2017 [30] de Moura et al., 2024 [31]	Direct scavenging of free radicals, inhibition of lipid peroxidation chain reactions	
Lai et al., 2016 [16]	Mitigate oxidative stress, reducing inflammatory markers	Antidiabetic activity
Tang et al., 2019 [32]	May inhibit α-amylase and α-glucosidase enzymes	
Tolmie et al., 2023 [34]	potentially slowing carbohydrate digestion	
Chen et al., 2012 [15]	Modulation of Gut Microbiota, alteration of microbial metabolite production, potential influence on energy production and fat storage	Anti-obesity activity
Kilel et al., 2013 [21]	Enhancing Triglyceride breakdown	
Kusano et al., 2008 [35]	Reduces fat absorption in intestines	
Yang et al., 2014 [36]	Inhibition of adipogenesis via downregulation of PPARγ and C/EBPα	
Chang et al., 2019 [37]	Increased thermogenesis through UCP1 expression in brown adipose tissue Potential modulation of appetite signals	
Rashid et al., 2014 [41], Novilla et al., 2017 [43], Bastos et al., 2009 [44], Lin et al., 2022 [45]	Inhibition of pro-inflammatory cytokines, modulation of NF-κB signaling pathway, reduction of oxidative stress-induced inflammation	Anti-inflammatory activity
Stangl et al., 2006 [46] Laudani et al., 2023 [47]	Anthocyanins improve endothelial function and promote vasodilation, potentially reducing hypertension	Cardiovascular benefits

Cardiovascular benefits

Numerous studies have been conducted on flavanols and anthocyanins from purple tea regarding their role in modulating or reducing risk factors and preventing cardiovascular health problems through various aspects of efficacy in vascular health including platelet aggregation, atherosclerosis, blood pressure, antioxidant status and inflammation markers. According to observational and interventional research, this wellness benefit occurs due to tea’s antioxidant content, mostly flavonoids. Tea and its bioactive components target Nrf2 activation and NF-kB inhibition is aimed at by these bioactive components [46]. There are high levels of polyphenols, especially anthocyanin content, making purple tea exhibit strong antioxidant properties. These agents help to decrease oxidative stress within the cardiac system, leading to a decreased risk of heart ailments [19,47]. Consumption of purple tea has been associated with improvements in lipid profiles. Research demonstrates that it may reduce the amounts of total and low-density lipoprotein (LDL) cholesterol while increasing high-density lipoprotein (HDL) cholesterol, thereby minimizing atherosclerosis risk. Studies have confirmed that drinking purple tea regularly is beneficial as far as blood pressure is concerned. The anthocyanins present in purple tea can increase the function of the endothelium as well as vasodilation, hence improving the flow of blood and reducing hypertension cases [47]. It has broad therapeutic spectra towards various cancers, synergism with some drugs used clinically against cancers, potent cardio-protective agents, psoriasis, and osteoporosis effects. Strong evidence from several research investigations supports numerous health benefits attributed to anthocyanins. These advantages include anti-cancer activities, cardiovascular improvement, and antioxidant activity, among others [9,29,48].

Therapeutic potential in obesity-related disorders

Polyphenols help to maintain a healthy intestinal microbiota by influencing the digestion of carbohydrates, uptake of glucose, and gluconeogenesis. Also, they aid in mitigating postprandial hyperglycemia symptoms through adipogenesis inhibition, prevention of lipogenesis, and stimulation of lipolysis/beta-oxidation that enhances lipid metabolism [5]. Many studies have shown that consuming foods high in anthocyanins can alleviate inflammation in adipose tissue as well as obesity-related dysbiosis in the gut. Among the various health benefits associated with anthocyanin is its ability not only to prevent diabetes but also to combat obesity, inflammatory bowel complications, and cancer [41-48]. Purple tea extract significantly suppressed a postprandial blood glucose rise among healthy individuals during a type 2 diabetes management study; however, Liu et al. had already established links between drinking tea and reducing incidences of type 2 diabetes mellitus [49]. Additionally, it promotes heart health because purple tea extract lowers cardiac risk factors such as blood pressure in hypertensive rats, while Shimoda et al. [5] demonstrated body weight reduction, BMI decrease, and body fat mass reduction in humans. In mice, PTE suppressed body weight gain, reduced fat accumulation, enhanced liver CPT1A expression, decreased liver weight and triglycerides, and inhibited fat absorption [5]. A study observed that tea catechins can help prevent and treat (NAFLD) Non-alcoholic fatty liver disease [50]. In another research work, it was found that purple tea has anti-inflammatory properties, which may help to manage chronic low-grade inflammation often seen in obesity. Purple tea is rich in antioxidants that can prevent oxidative stress associated with obesity [19]. The researchers showed that purple tea extract improved cognitive function in a mouse model of (AD) Alzheimer’s disease [51]. Limited research has been done on purple tea and cancer, but CS Yang et al. delved into the role of tea polyphenols in the prevention and treatment of cancer [52]. According to the authors, osteoarthritis a common complication of overweightness could be mitigated by tea polyphenols through their anti-inflammatory and antioxidative mechanisms [53]. These phytochemicals lower obesity through the modulation of various mechanisms such as energy intake, expenditure, preadipocyte differentiation, and proliferation, among others. Accordingly, these phytonutrients have been formulated into innovative food products to reduce obesity prevalence and enhance public health status. However, one must consider that even if some studies look promising, they are mainly preclinical or small-scale human trials that need wider clinical trials on large groups before determining how effective yellow tea is at managing comorbidities associated with excessive weight gain. Therefore, more investigations should be made regarding appropriate dosages of yellow tea for these conditions, as well as its safety issues over long periods.

Potential synergistic effects of exercise

Anthocyanins are polyphenols contained in purple tea, and they facilitate lipid oxidation. This can be increased by exercising. Green tea catechins, including EGCG, promote thermogenesis. They help to increase energy expenditure within one day for green tea extracts. The presence of catechins and other bioactive compounds makes purple tea a possible catalyst of thermogenesis [37]. It may, therefore, be used as an agent of fat burning, leading to lower calorie uptake that could assist in managing weight effectively when exercising [54]. Drinking purple tea and regular exercise have been associated with improved cardiovascular health. There is a possibility of this effect being additive or synergistic. Researchers found out that those who drank green tea daily had fewer chances of mortality because of cardiovascular problems. Purple tea drinking might also provide increased protection against heart disease when combined with the known cardioprotective benefits provided by exercise [55]. A certain study claimed that moderate exercise could boost fat oxidation, especially if green tea extract were used together with it. On the other hand, since there can be several similarities between the two types but have different elements, equivalent or superior actions may be expected from purple tea, hence the potential for better body composition [56].

Regular exercises tend to improve insulin sensitivity and glucose metabolism more than anything else does. Such an advantage may also exist in purple tea habituation, where they will operate together more efficiently. In conjunction with this, there is evidence from recent research demonstrating that green tea extract administration plus exercises enhanced glucose tolerance in obese mice far better than alone. The ability of purple teas to regulate glucose metabolism clearly shows how they can work together with exercise, thus improving metabolic health [57]. A synergistic effect on general health, specifically cardiovascular fitness, metabolic health, and parameters of exercise, has been observed among individuals who drink purple teas in addition to regular physical activities. Given the lack of research on purple tea and exercise, conclusions may be drawn from other tea types based on purple tea’s distinctive composition. Antioxidants and anti-inflammatory components of purple teas may assist in recovery from training. Furthermore, a study found that tart cherry juice rich in anthocyanins, like those present in purple teas, significantly improved recovery from exercise-induced muscle injury [58]. Tea catechins are linked to enhanced endurance performance in animals and promote fat oxidation during cycle ergometer exercise in adult humans [59]. The distinct polyphenol profile of purple tea, including GHG, catechins, and anthocyanins, could be equally or more advantageous for regular exercising endurance athletes. Neuroprotective properties are possessed by Tea Polyphenols [60]. Combining Purple tea intake with steady training may confer significant cognitive benefits, such as increased concentration and better mental performance during workouts. The most recent research delved into the effect of a short oral dosing interval using purple tea on humans following intense trauma exercise. The researchers concluded that acute purple tea supplementation decreased lactate dehydrogenase, a muscle damage marker, but improved lower limb muscle endurance performance [11]. These potential combined effects are interesting; however, more studies are necessary, especially on purple tea synergy with working out.

Safety and potential side effects

Although generally safe for most people, purple tea may have potential side effects and safety concerns, particularly when eaten in excessive amounts or by some individuals. Purple tea leaves contain several bioactive compounds, with principal constituents including theobromine (1.6%), caffeine (4.4%), EGCG (epigallocatechin gallate, 9.8%), GHG (1,2-di-Galloyl-4,6-Hexahydroxydiphenoyl-β-D-Glucose, 7.4%), and ECG (epicatechin gallate, 5.8%), with these compounds showing seasonal variations throughout the year [5]. Like other *Camellia sinensis *teas, purple contains caffeine but in lesser quantities compared to green or black teas. According to Kerio et al., purple had approximately 1.8-2.5% caffeine levels based on dry weight basis. Too much caffeine can cause sleeplessness, increased heart rate and blood pressure, anxiety as well as digestive problems [19]. Some polyphenols in teas, like purple tea, can interfere with iron absorption, which might be of concern to those at risk of iron deficiency [61]. It is important to note that there is limited research investigating the safety profile of purple tea; hence, some information is derived from studies done on other types of teas. Although less acidic than other drinks, tannins in purple tea may discolor teeth over time [62]. Tea contains oxalates, which can cause kidney stone formation in some individuals at risk for kidney stones [63]. Some drugs like anticoagulants and iron supplements can be affected by the catechins. For this reason, pregnant women who are using tea need to be cautious because of its caffeine content [64]. While high levels of antioxidants in purple are generally healthy, excessive intake of antioxidants could have adverse effects [65]. Purple tea, like other teas, may contain pesticide residues if it’s not organically grown, and heavy metals from the soil might be absorbed by tea plants. A few people may experience stomach pain and headaches after consuming tea without eating food first or when they drink a lot of tea together. Tea that is improperly stored or handled can be contaminated with microorganisms [66]. Most of these potential side effects are linked to overuse or apply to specific groups. Overall, well adults can safely enjoy moderate amounts of purple tea; on the contrary, caution needs to be taken by those with pre-existing health conditions or taking medication, as well as pregnant or nursing women about frequently ingesting purple tea. Additional studies, therefore, should focus on extracting individual bioactive compounds to identify highly active substances that can be used maximally as chemotherapeutics.

Clinical studies

Richards et al. conducted a study using green tea extract to show the potential effects of tea catechins on exercise performance, although it is not about purple tea [59]. In this randomized, placebo-controlled study, healthy adult participants experienced an increase in maximal oxygen consumption and exercise performance following the administration of green tea extract supplements. However, further studies may provide similar findings, given that purple tea contains high levels of catechin and is associated with exercise. The study by Shimoda et al. demonstrated that mice receiving purple tea extract (PTE) at 200 mg/kg experienced significant reductions in body weight, liver weight, abdominal fat, and triglycerides, coupled with increased CPT 1A protein expression [5]. A subsequent four-week human trial revealed statistically significant improvements in obesity metrics: body weight decreased from 79.9±3.1 kg to 80.8±3.2 kg (p<0.05), body mass index from 26.8±0.6 to 27.0±0.6 (p<0.05), and body fat mass from 21.0±1.4 kg to 21.8±1.5 kg (p<0.01). These findings suggest PTE's potential to mitigate diet-induced weight gain by suppressing fat absorption and enhancing hepatic fat metabolism, potentially through the synergistic effects of caffeine, catechins, and the unique compound GHG prevalent in purple tea leaves [5]. An initial clinical investigation assessed the acute effects of consuming purple tea on cognitive performance and mood swings. This consisted of a small, randomized crossover study with 20 healthy adults aged between 18 and 30 years old. Findings show that drinking purple tea may enhance certain aspects like attention and mood, but further investigations are required to confirm these claims [67]. A randomized, double-blind, placebo-controlled cross-over trial examined whether brief oral dosing with purple tea was efficacious on treadmill exercise in humans after stresses which damaged muscle tissue. The study involved thirty healthy people who undertook a brief bout of high-intensity aerobic exercise followed by intermittent resistance training using their thigh muscles only. Acute supplementation with purple tea decreased lactate dehydrogenase levels, suggesting reduced muscle injury while enhancing lower-body muscle endurance [11]. Nevertheless, it is important to note that these studies present encouraging results; however, more comprehensive long-term clinical trials should be conducted for a complete understanding of the health-promoting properties possessed by purple tea. Furthermore, additional research should be conducted to establish an optimal dosage range and long-term safety profile for the use of purple tea.

Potential interactions with medications

Purple tea contains bioactive molecules as with other teas from *Camellia sinensis* and thus has potential interactions with medications or supplements consumed alongside it. Although limited data exist concerning interactions involving purple tea, studies on green teas and black teas can provide useful insights. Apart from reducing iron absorption due to polyphenols contained in tea, these interactions also include vitamin K contents that may interfere with anticoagulants like warfarin, caffeine’s propensity to enhance stimulant medications’ effects, the possibility of enhancing antihypertensive and antidiabetic drugs, and possible interactions with tyramine content due to (MAOI) monoamine oxidase inhibitors [68]. For example, oxalates can impair calcium absorption, disrupt folate metabolism, impair the effectiveness of some antibiotics, affect the pharmacokinetics of antipsychotic drugs, and interact with certain chemotherapeutic agents [63,69]. The severity and clinical relevance of such interactions differ largely between patients as they depend on various factors, including individual characteristics, dosage, and frequency. Furthermore, individuals who use medications or supplements should avoid large amounts of tea that do not alter intake patterns or administration times relative to drug consumption. Recent research studies suggest that it is unlikely that green tea extract or its constituent catechins would have a clinically meaningful effect on major cytochrome P450 or UDP-glucuronosyltransferase (UPDGT) enzyme substrates or agents functioning as P-glycoprotein substrates. However, considerable doses of green tea drinks or extracts should be used cautiously in patients prescribed recognized substrates of organic anion transporting polypeptide, especially those with a narrow therapeutic index [70]. The impairment in digestion enzymes is caused by polyphenol-protein complexes. This ability will either increase enzymatic activity or have inhibitory action on enzymes depending on how proteins can interact with polyphenols during digestion. It might inhibit CYPs, thereby affecting the oral bioavailability of a drug [71]. Even though these are based mainly on studies involving black teas and green ones, besides theoretical assumptions about known compounds present in various brands, they offer valuable information when one is consuming purple tea with medications and supplements [9].

Comparison with other weight loss interventions

Purple tea, which contains unique polyphenols and anthocyanins, has been attracting attention as a possible way to lose weight. Starch digestion is inhibited by purple tea more than other aqueous tea extracts due to its action on pancreatic α-amylase. This demonstrates a greater potential for after-dinner hypoglycemic effect mainly due to the presence of polyphenols and anthocyanins, and purple tea proved to be more sensitive than green tea Table 2. The study showed PTE's effectiveness in reducing body weight, liver weight, abdominal fat, and triglycerides in mice, with increased carnitine palmitoyl transferase (CPT) 1A protein expression. A subsequent human trial demonstrated statistically significant improvements in obesity metrics. The findings suggest PTE's potential to mitigate diet-induced weight gain by suppressing fat absorption and enhancing hepatic fat metabolism [5]. Purple tea shows promise but offers less impressive results than other treatments for obesity. A meta-analysis showed that green tea, which was also a popular tool for weight reduction, had similar small beneficial effects on both weight loss and maintenance [72]. This means that calorie restriction often causes between 5-10% weight loss in 6 months compared to that achieved from tea alone [73]. The study highlighted that exercise programs resulted in as much weight loss as those involving the consumption of teas when assessed independently, but such combinations improved the ability of someone to shed some pounds [74]. Most pharmacological interventions result in about 3-9% more weight reduction compared to what is observed with placebo subjects only or those using certain types of teas, including purple ones [75]. Concerning nutritional supplements, there may be stronger indications for the use of purple tea. Many dietary supplements used for slimming down have not been scientifically proven. Intermittent fasting has been reported as causing up to 3-8% weight loss in 12 weeks, significantly greater than for purple tea only. The differences were, however, non-significant at twelve months. Low-fat diets led to greater short-term weight reductions compared to low-carbohydrate diets, according to a research meta-analysis; however, there were no significant differences after one year [76]. The best thing about purple tea is that it can be easily incorporated into everyday life, and it has the potential for other health benefits as well, beyond just weight loss, such as anti-oxidative effects and cardiovascular benefits. However, most successful weight loss therapies employ a combination of measures. Instead of being a solution on its own, purple tea may be an important component of an overall plan for healthy weight reduction. However, little research has been conducted in this respect, especially in long-term studies on purple tea for slimming purposes. Additionally, more research comparing its effectiveness to different modes of therapy over lengthy durations and across populations is needed. Besides, the right dose of purple tea for losing weight remains unknown, while future investigations should focus on possible long-term negative effects associated with such interventions.

**Table 2:** Comparison of purple tea with other teas. GHG: 1,2-Di-Galloyl-4,6-Hexahydroxydiphenoyl-β-D-Glucose.

Author, Year	Health Benefit/Variation	Purple tea	Other teas	Advantages of purple tea
Lai et al., 2016 [16]	Antioxidant activity	Higher total antioxidant activity due to flavonoids and anthocyanins	Green and black teas have lower antioxidant activity	Superior antioxidant properties
Kerio et al., 2013 [19]	Caffeine content	Generally, lower caffeine content	Green and black teas typically have higher caffeine	A better option for lower caffeine lovers
Kilel et al., 2013 [21]	Diabetes prevention	It contains a unique compound, GHG, with anti-diabetic characteristics	Other teas lack GHG	Potentially better for diabetes prevention
Kilel et al., 2013 [21]	Unique compounds	High in anthocyanins (delphinidin and cyanidin glycosides) and GHG	Other teas lack significant anthocyanin content and GHG	Unique health benefits associated with these compounds
Ahmed et al., 2013 [23]	Flavor profile	Milder, smoother flavor with delicate sweet notes	Green tea: grassy, vegetal; Black tea: strong, potentially acrid	More palatable
Tolmie et al., 2023 [34]	Starch digestion inhibition	More effective in inhibiting starch digestion	It is less effective than purple tea	Greater potential for hypoglycemic effect
Stangl et al., 2006 [46] Pascual-Teresa De et al., 2010 [48]	Cardiovascular health	Potential cardiovascular benefits	Black tea may offer protection from heart disease	Similar cardiovascular effects with increased oxidative protection
Hursel et al., 2009 [72]	Weight management	Significantly reduced body weight, BMI, and visceral fat in mildly obese individuals	Green tea shows similar small beneficial effects	Potentially more effective for weight loss

Synergistic combinations

The study was conducted in Rwanda and Kenya and involved the selection of superior tea clones with high catechin content from known tea clones, exploring their antiproliferative effects on the triple-negative breast cancer cell line (TNBC) as well as determining their combination index with cisplatin. The findings revealed that catechin extracts from purple tea exhibited better anti-proliferative activity against TNBC cells combined with cisplatin [77]. Anthocyanins present in the purple tea leaves are strong antioxidants that may influence improved bioactivity. These may act together to increase effectiveness, perhaps enhancing overall antioxidant activity [42]. Hence, there is potential for synergy between teas and pharmaceuticals to enhance positive health outcomes. Purple tea has unique phytochemicals, such as GHG, that can form novel synergistic relationships with specific drugs [78]. It was observed that consuming purple tea leaves (3 gm/day) over a period of four weeks improved obesity indices in human volunteers. This implies that purple tea drinking may have some complementary impact on dietary interventions in weight loss programs [5]. On the other hand, it has been shown in numerous scientific studies that combining different types of teas with herbal medicines can be helpful by causing synergism between them to improve the level at which they can fight diseases like diabetes and cancer or just act as general antioxidants [16]. Its specific composition is made up of GHG and anthocyanidins, with lower levels of caffeine, which might offer additional benefits when combined with herbs [19]. While these promising synergistic combinations are seen, more research is required to fully understand and assess their benefits and potential uses in health promotion and disease prevention.

Commercial products of purple tea

There are several commercial products, but most do not indicate the actual percentages of bioactive ingredients. Purple tea products are on sale at JusTea, WSTEA, Zija International, the Republic of Tea as well as Teaspec, emphasize high antioxidant content, mostly in terms of anthocyanins and polyphenols, although the exact proportions remain undisclosed. Scientific studies provide more definite information about purple tea’s composition. A study revealed that the total polyphenol content ranged from 17.1-21.1% in aerated and 18.8-24.6% in unaerated teas processed from purple leaf cultivars [19]. Likewise, Kilel et al. found that it has 12.6-17.1% total polyphenols and 0.05-0.2% anthocyanin ranges [21]. Such variations can be attributed to differences in growing conditions, processing techniques as well as cultivars used. Certain standardized purple tea extracts such as Porelis™ (Vidya herbs) contain around 3-5 % GHG. Alluvia™, a standardized extract, contains approximately 45-50% polyphenols, 9-10% EGCG, and 1-1.5% anthocyanins. It also includes purple tea standardized extracts combined with alpha-linolenic acid, docosahexaenoic acid (DHA), and gamma-linolenic acid, produced by Oryza Oil & Fat Chemical Co. Limited. Alpine Bliss™ purple tea slimming formula, while not a pure extract, incorporates Alluvia® purple tea extract as part of its proprietary ThermoBliss™ blend. This product claims to have 16-16.5% polyphenols in the purple tea component. Other brands are Sunphenon purple tea extract (Taiyo International), purple tea CG (Changsha Herbway Biotech), and Finlay’s GR purple tea extract, which also offer unique standardizations based on customer needs. If compound ratios are not included, consumers should go for brands that encourage transparency in sourcing and processing when choosing purple tea. Also, the extraction process utilized and the individual components being standardized should be considered when selecting a standardized extract [5].

Opportunities and challenges

Purple tea and the knowledge gaps are future areas of study that offer exciting opportunities for scientific exploration. This unique type of tea, which was mainly developed in Kenya, has raised curiosity due to its potential health benefits as well as its peculiarities. While the pigment content of purple tea is known, a more comprehensive analysis of its phytochemical constitution is needed. Further research should aim to identify and quantify all bioactive compounds, including lesser-known flavonoids, catechins, and other polyphenols found in purple tea. Future studies may adopt more advanced analytical techniques with greater resolution capabilities for better precision in the composition and concentration of various flavonoids present in tea samples. The human body's absorption systems and how they metabolize some of these ingredients remain a big part of lacuna. Anthocyanin bioavailability and related metabolic pathways should be covered by further research on purple tea’s contents in human beings. No long-term, large-scale clinical trials have been conducted, but different research suggests that purple tea might be beneficial for health purposes. Robust randomized controlled trials need to be considered in future research aimed at determining whether regular consumption of purple tea has any impact on such health outcomes as heart disease risk factors, e.g., lipid profiles; prognostic indicators like hsCRP; or biomarkers involved with cognition (e.g., dopamine). It is necessary to acquire additional data regarding the purported health benefits associated with drinking purple rather than green/black/oolong teas. In this regard, this paper will help to place purple tea within a broader context of the overall pattern and consequences of drinking teas. Furthermore, this study would also search for genetic pathways leading to the production of anthocyanin pigments, resulting in purple coloration and specific chemical composition, among other variations observed in purple tea.

Moreover, a study examining the impact of environmental factors (e.g., soil type, climate, and altitude) on phytochemical levels in tea might yield insights into better cultivation techniques. Little is known about the effect of different processing methods and preparation techniques on bioactive constituents in purple tea. There should be further research to identify how different procedures for preparing or processing change nutrition as well as the functional properties of this drink. Purple tea has potential synergistic interactions with other nutrients or medications that could be fascinating to explore. This would be highly applicable to functional foods or nutraceuticals. Although purple tea is generally believed to be safe, there exist few comprehensive toxicologic and safety studies, especially those that use concentrated extracts or high doses. Future studies should create bridges that will help attain safe intake limits. More research is needed to find out if purple tea is economically viable, what its environmental consequences are, and what sustainable agricultural practices are applied hereto. Such investigations may provide useful ways to achieve sustainability within the purple tea production sector. Future research should focus on other uses of purple tea apart from just drinking it as a regular beverage; such uses can include functional foods, cosmetics, and natural food colorants, among others. A study examining possible epigenetic effects of taking purple tea could elucidate how it alters gene expression, thus affecting long-term health implications for human beings. As more attention grows toward a gut-brain axis relationship, investigating how purple tea components interact with and modify the gut microbiome may reveal novel health benefits. Addressing these research gaps and following these future approaches requires multidisciplinary collaboration. Such wide study efforts could considerably improve our understanding of purple tea, potentially leading to novel applications in nutrition, health, and beyond.

Future perspectives

More research needs to be performed to determine the synergistic effect of its beneficial ingredients and how they can be used together with exercise or other treatments. Nevertheless, there remains a large knowledge gap that necessitates further rigorous clinical trials over longer periods of time, extensive safety evaluations, and mechanistic investigations. As the study progresses further, it would be important to know the correct dosage, preparation techniques, and any possible interactions with medication or dietary supplements. Also, its usage as a tool for managing other diseases associated with obesity should be investigated before analyzing its efficacy compared to current weight loss methods. However, we must temper our excitement about purple tea's possibilities with careful scientific studies. Therefore, only by carrying out sustainable research will we be able to realize the full potential of purple tea through making recommendations based on evidence for use in promoting human health and wellbeing.

## Conclusions

Purple tea is emerging as a promising area in functional foods and nutraceuticals, particularly for weight management and health enhancement strategies. It possesses a unique phytochemical profile rich in anthocyanins, polyphenols, and GHG, setting it apart from other tea varieties. Initial assessments suggest potential effects on lipid metabolism, blood sugar regulation, antioxidant activity, and gut flora, although the evidence is currently weak and preliminary. There is a significant knowledge gap regarding purple tea's effects, and while it shows potential as an adjunctive therapy for obesity and metabolic syndrome, enthusiasm must be tempered with careful scientific scrutiny.
